# Micronutrient deficiencies in the elderly – could ready meals be part of the solution?

**DOI:** 10.1017/jns.2016.42

**Published:** 2017-01-12

**Authors:** Richard Hoffman

**Affiliations:** Department of Biological and Environmental Sciences, School of Life and Medical Sciences, University of Hertfordshire, Hatfield AL10 9AB, UK

**Keywords:** Micronutrients, Vitamins, Phytochemicals, Elderly, Western diet, Ready meals, Ready-to-eat foods

## Abstract

Micronutrient deficiencies contribute to many age-related disorders. One group at particular risk of micronutrient deficiencies is the elderly. Many elderly, such as the frail and those living in institutions, rely on ready meals of variable, often poor, nutritional quality for a significant part of their daily nutritional needs. New policies are needed to ensure that micronutrients (vitamins and minerals) and phytochemicals of known nutritional value are retained during the manufacture of ready meals. This together with increased awareness of the importance of micronutrients for health, and simple, clear labelling of the micronutrient content of ready meals would help in the choice of healthier products. Professionally prepared ready meals monitored by nutritionists and dietitians can help achieve these goals so that ready meals become part of the solution to poor nutrition in the elderly, rather than being viewed as part of the problem.

Despite extensive academic research into the causes and consequences of suboptimal nutrition, many groups in society continue to eat a poor diet. The Global Burden of Disease study found that in the UK, poor diet is now the leading contributor to disability-adjusted life years (DALYs) – an estimate of premature mortality and non-fatal health loss^(^^1^^)^. Suboptimal intake of micronutrients (vitamins and minerals) and antioxidant and anti-cancer phytochemicals is a major contributor to DALYs, and is linked to many age-related disorders including CVD, some cancers and Alzheimer's disease^(^^2^^,^^3^^)^. Hence, there is an urgent unmet need to ensure adequate micronutrient intake.

It is widely agreed that cooking from raw ingredients is the best way to achieve a balanced diet. However, some sectors of society, such as many frail elderly, cannot cook for themselves. Instead, they rely on suppliers of ready meals (defined as pre-prepared main courses reheated in their container, requiring no further ingredients and minimal preparation^(^^4^^)^) to provide their main meal of the day. There is currently very little regulation on the micronutrient content of ready meals and equally little information on their micronutrient content – either for the consumer or in the scientific literature. And yet micronutrient deficiencies are widespread in the elderly^(^^5^^)^. Hence, not knowing the micronutrient content of ready meals is a concern for the health of elderly people reliant on these meals.

Ready meals are often viewed as part of the problem of poor nutrition – a reflection of their variable, often poor, nutritional quality. So what is needed to instead make these meals part of the solution to micronutrient deficiencies for those who rely on them for a large part of their daily nutritional requirements? Should there be more obligations on manufacturers and providers (retailers, care homes, hospitals or providers of meals on wheels) to ensure that ready meals retain the micronutrient and phytochemical content of their raw ingredients? And how can consumers be made more aware of the micronutrient content of ready meals and the importance of micronutrients in their diets so that they demand better-quality products?

## Micronutrient deficiencies in the elderly

The current perspective on diet-related diseases mostly focuses on obesity and obesity-related diseases. However, this emphasis on reducing energy intake risks deflecting attention from the serious problem of malnutrition. About 15 % of UK public spending on health and social care – estimated at £19·6 billion in 2011–2012 – is to treat malnutrition, and about half of this expenditure is directed at older people (>65 years)^(^^6^^)^. Although the main focus of malnutrition in the elderly is on protein–energy undernutrition, especially in the very old (75+ years), the elderly as a whole are also frequently micronutrient deficient. A recent National Diet and Nutrition Survey (NDNS) in the UK reported that dietary intakes of vitamin D, K, Mg and Se in the elderly living in the community were frequently below recommended nutrient intake values^(^^7^^)^. Also, a systematic review of observational cohort and longitudinal studies on the habitual intake of community-dwelling older adults (≥65 years) in Western developed countries concluded that intakes of vitamin D, thiamine, riboflavin, Ca, Mg and Se were of concern^(^^5^^)^.

Some sectors of the elderly population are particularly vulnerable to micronutrient deficiencies and this may be under-reported in more generalised surveys of the elderly living in the community (such as the NDNS). Living in an institution, being malnourished or frail, or being overweight (in the UK, 78 % of men and 71 % of women aged 65–74 years are overweight or obese) all increase the risk of micronutrient deficiencies^(^^8^^–^^10^^)^. In addition, increasing numbers of elderly are being diagnosed with coeliac disease and this is linked to an increased risk of micronutrient deficiencies. These deficiencies may arise because coeliacs avoid fortified wheat products^(^^11^^)^, or because of malabsorption of micronutrients (including Fe, Ca, folate, and fat-soluble vitamins) during the average time to diagnosis of 14 years^(^^12^^)^.

The initial effects of micronutrient deficiencies may be relatively mild, diffuse and subclinical and hence easily missed. For example, deficiencies in B vitamins may result in mild cognitive decline^(^^13^^)^; thiamine insufficiency increases levels of advanced glycation end-products^(^^14^^)^ (which are linked to the development of type 2 diabetes); deficiencies of vitamin B_12_ and folate raise homocysteine levels that are linked to CVD, and a lack of vitamin D changes immune function. Prevention of micronutrient deficiencies is key to successful management, a policy that would bring significant savings compared with the cost of treating diseases arising from malnutrition^(^^6^^)^.

## Consumption of ready meals by the elderly

Achieving a balanced nutritious diet cooked from raw ingredients is a challenge for the large swathes of the UK population for whom home cooking is simply not popular. Moreover, healthy eating campaigns, like the National Health Service's Change4Life, are not having much success^(^^15^^)^. Hence, many turn to ready meals. Data from the Department for Environment, Food and Rural Affairs (DEFRA) show that between 1974 and 2014, vegetable-based ready meal consumption in the general population increased from 8 to 50 g per week, and meat-based ready meals from 5 to 85 g per week^(^^16^^)^.

This move to ready meals includes many of the 11·4 million elderly in the UK. Frailty or lack or loss of a spouse, especially among elderly men, can reduce the incentive and ability to cook^(^^17^^)^. Hence, it is not surprising that the elderly are major consumers of ready meals, whether bought from retailers or supplied by community delivery services such as ‘meals-on-wheels’. One in twelve older people in the UK is estimated to receive a community meal at home and these are frequently the main meal of the day. In addition, according to Age UK, approximately 405 000 UK elderly live in residential care homes where most receive their meals from providers.

## Making ready meals part of the solution

Current dietary advice to the general population is usually to limit consumption of ready meals since many are high in saturated fat and salt (although not necessarily in sugar and total energy)^(^^4^^)^. By contrast, information on the micronutrient content of ready meals is limited to a few reports, such as the folate content of some Spanish ready meals^(^^18^^)^ and some limited information in McCance and Widdowson^(^^19^^)^. Hence, it is currently not possible to determine to what extent a diet high in ready meals is contributing to diseases associated with micronutrient deficiencies. Nevertheless, ensuring that these meals provide levels of micronutrients commensurate with their raw ingredients is essential for the many people who rely on them.

The switch of food production from the home kitchen to external meal providers presents not only the threat of nutritionally poor meals, but also opportunities. An important source of micronutrients is vegetables, and yet these are low in many popular ready meals such as roast beef dinner and cottage pie. But the skills of the food industry's chefs could be used to provide tasty, desirable alternatives higher in vegetables and micronutrients. It is especially important to increase consumption of green leafy vegetables since these are a major dietary source of folates and Mg, both frequently deficient in the elderly. They are also an excellent source of lutein, a carotenoid associated with improved cognition in the elderly^(^^20^^)^.

Professionals have the skills to produce tasty ready meals from plant-based cuisines such as the Mediterranean diet – something often beyond the culinary skills of elderly households. The Mediterranean diet has been shown to be particularly effective at restoring recommended levels of micronutrients^(^^21^^)^. It also reduces the risk of age-related diseases such as Alzheimer's disease. As Alzheimer's disease is linked to multiple micronutrient deficiencies, it is not surprising that the micronutrient-rich Mediterranean diet is far more effective than any single supplement at reducing the risk of Alzheimer's disease^(^^22^^)^.

Professionally prepared ready meals monitored by nutritionists and dietitians can also ensure that losses of micronutrients are minimised. There is a wealth of evidence for food production processes reducing micronutrient levels. (1) Heat inactivates some vitamins. (2) Cooking vegetables in excess water causes leaching of water-soluble vitamins and phytochemicals such as anti-cancer glucosinolates and some phenolics^(^^23^^)^. (3) Increases in pH – such as when bicarbonate is used to retain the colour of green vegetables or help soften pulses – can destabilise vitamins such as thiamine, pantothenic acid, vitamin C and some folates^(^^24^^)^. (4) Use of sulphite as a preservative can destroy thiamine. Although sulphite is banned in some countries in products that are a significant dietary source of thiamine, it is widely used in the UK to preserve tinned pulses, convenience meat products (such as sausages, prepared meat balls and burgers), and many ready meals and convenience foods that contain potatoes. Its effects on thiamine levels can be dramatic: whereas a grilled pork chop is an excellent source of thiamine (0·78 mg/100 g), grilled sausages contain only trace amounts^(^^19^^)^. Pork sausages frequently replace pork meat in ‘meals on wheels’; so removing sulphites would be a straightforward way to improve nutritional value without needing to change diet.

Using traditional culinary practices can be a practical way to help preserve micronutrients during the production of ready meals. For instance, traditional Mediterranean cuisine frequently stews vegetables rather than boiling them: this retains micronutrients that would be lost by leaching. And cooking at low temperatures reduces nutrient losses due to heat. With guidance from dietitians and nutritionists to minimise micronutrient losses and a new nutritional standard for ready meals, a slogan like ‘Vegetables slow-stewed for health’ could become as effective a marketing tool as currently popular slogans like ‘Wood-fired pizzas’ or ‘Free-range chicken’.

## Retaining micronutrients during production is preferable to enrichment

Ready meals coated with a thin layer of gelatin fortified with vitamin D_3_ and Ca are being developed for the elderly^(^^25^^)^. But enrichment with specific micronutrients cannot fully compensate for the myriad of micronutrients, phytochemicals and fibre that can be reduced when food is prepared without due care. For example, although fortified cereals are a significant source of micronutrients for some sectors of the UK population, reconstructing refined cereal products by adding back a handful of micronutrients (Ca, Fe, niacin and thiamine) to compensate for those lost during refining does not restore the many other vitamins, minerals, phytochemicals and fibre that were present in the germ and bran^(^^26^^)^. The importance of phytochemicals is increasingly recognised: some have been termed ‘lifespan essentials’ needed for optimal health^(^^27^^)^. For example, lutein is the main carotenoid in the brain where it may reduce the risk of Alzheimer's disease^(^^28^^)^, some polyphenols reduce glucose uptake and so may lower the risk of type 2 diabetes^(^^29^^)^, while certain flavonoids improve vascular function^(^^30^^)^. Additionally, the importance of synergies between nutrients is increasingly recognised, and phytochemicals can synergise with vitamins, as in the interactions between carotenoids and vitamin E as antioxidants^(^^31^^)^.

Hence, it is now generally agreed that the optimal diet is based on a variety of unrefined foods, including a wide variety of plant foods that provide the dietary fibre and phytochemicals often lacking in the typical animal product-orientated Western diet. This ‘whole-diet’ approach was endorsed in a recent consensus statement by a panel of leading nutritionists convened by the Oldways Organisation^(^^32^^)^, in a report on the European Union (EU) elderly^(^^33^^)^, and forms the basis for dietary guidelines in many countries, including the 2015 US dietary guidelines. Diet is also the mainstay of the so-called Food First strategy in the UK for treating the malnourished elderly (with supplements used for acute micronutrient deficiencies).

## Providing better consumer information

In stark contrast to the mandatory requirement in the EU from December 2016 to label fat, sugar and salt on prepackaged foods, information on micronutrient content is infrequent (except on fortified cereal products), and the new EU legislation will not require this to change. Advice on food preparation and meal planning to provide adequate micronutrients is available to community catering organisations in the UK (for example, from the National Association of Care Catering and The Caroline Walker Trust), but much of this is voluntary.

Compositional data based on raw ingredients are usually acceptable for macronutrients, which do not change significantly during food production. But micronutrient content can change significantly during processing, so using data based on the raw ingredients may not be appropriate. Several databases, such as that from the US Department of Agriculture, provide retention factor values in order to help estimate the proportion of micronutrients that are retained after food preparation. Levels of polyphenols in some processed and cooked foods are also available^(^^34^^)^. However, these values can only provide an estimate of micronutrient content, due to wide variations in food processing techniques. The ‘gold standard’ will always be direct measurement of micronutrients in the final product.

Communicating the micronutrient content of ready meals to the consumer requires care. Although sophisticated nutrient profiling systems are being developed, these may not be best for engaging the elderly community. A simple quality marque may be better, similar to the UK Soil Association's organic marque. But first, public awareness of the importance of micronutrients must be raised. A recent Food Standards Agency survey found that although consumer awareness and concern for the sugar, salt and fat content of foods was high, micronutrients did not even get a mention^(^^35^^)^. One option would be to relate the micronutrient marque to a health claim, for example, ‘Contains micronutrients needed for brain health’, rather than stating the levels of specific micronutrients.

## Discussion

There is an urgent need to improve the micronutrient content of the diets of many vulnerable groups. Although cooking from raw ingredients is key to a healthy balanced diet, for some, ready meals are the only option. It is time to abandon accepting ready meals as products of low nutritional quality. Instead, nutrition scientists and dietitians, and manufacturers and providers of ready meals need to come together to ensure that ready meals are produced to high standards so that consumers are safe-guarded against micronutrient deficiencies.

For many, current policies to increase home cooking are failing, and there is always the danger that advocating ready meals for those who cannot cook risks further alienating others from home cooking. So new policies are also urgently needed that successfully promote and facilitate the social and recreational benefits that come from buying and preparing food for home cooking, as well as the health benefits. This approach will help ensure that cooking skills are passed from one generation to the next, lessening the risk of future generations developing ever more reliance on commercially produced meals.
